# Supplementary material for: Bayesian refinement of protein structures and ensembles against SAXS data using molecular dynamics
Source: PLoS Comput Biol. 2017 Oct 18;13(10):e1005800. doi: 10.1371/journal.pcbi.1005800 (PMC5662244; doi:10.1371/journal.pcbi.1005800)
Supplement: S1 Archive — (BZ2) [file pcbi.1005800.s011.tar.bz2 › gromacs_4.62_bayesian_saxs_refi/share/html/online/g_anadock.html]

g\_anadock


| g\_anadock Main Table of Contents |

**VERSION 4.6  
Sat 19 Jan 2013**

---

### Description

g\_anadock analyses the results of an Autodock run and clusters the
structures together, based on distance or RMSD. The docked energy
and free energy estimates are analysed, and for each cluster the
energy statistics are printed.

An alternative approach to this is to cluster the structures first
using g\_cluster and then sort the clusters on either lowest
energy or average energy.

### Files

| option | filename | type | description |
| --- | --- | --- | --- |
| **-f** | eiwit.pdb | Input | Protein data bank file |
| **-ox** | cluster.pdb | Output | Protein data bank file |
| **-od** | edocked.xvg | Output | xvgr/xmgr file |
| **-of** | efree.xvg | Output | xvgr/xmgr file |
| **-g** | anadock.log | Output | Log file |

### Other options

| option | type | default | description |
| --- | --- | --- | --- |
| **-[no]h** | bool | no | Print help info and quit || **-[no]version** | bool | no | Print version info and quit || **-nice** | int | 0 | Set the nicelevel || **-xvg** | enum | xmgrace | xvg plot formatting: xmgrace, xmgr or none || **-[no]free** | bool | no | Use Free energy estimate from autodock for sorting the classes || **-[no]rms** | bool | yes | Cluster on RMS or distance || **-cutoff** | real | 0.2 | Maximum RMSD/distance for belonging to the same cluster |

---

http://www.gromacs.org  
gromacs@gromacs.org
